# Comparative Anatomy of the Optic Nerve Head and Inner Retina in Non-Primate Animal Models Used for Glaucoma Research

**DOI:** 10.2174/1874364100802010094

**Published:** 2008-05-09

**Authors:** Christian Albrecht May

**Affiliations:** Department of Anatomy, Medical Faculty Carl Gustav Carus, Technical University Dresden, D-01307 Dresden, Germany

**Keywords:** Morphology, lamina cribrosa, optic nerve head, inner retina, animal models.

## Abstract

To judge the information of experimental settings in relation to the human situation, it is crucial to be aware of morphological differences and peculiarities in the species studied. Related to glaucoma, the most important structures of the posterior eye segment are the optic nerve head including the lamina cribrosa, and the inner retinal layers. The review highlights the differences of the lamina cribrosa and its vascular supply, the prelaminar optic nerve head, and the retinal ganglion cell layer in the most widely used animal models for glaucoma research, including mouse, rat, rabbit, pig, dog, cat, chicken, and quail. Although all species show some differences to the human situation, the rabbit seems to be the most problematic animal for glaucoma research.

Glaucoma is a chronic disease appearing in a number of different conditions that are merged pathologically by the degeneration of retinal ganglion cells and their processes within the optic nerve and clinically by specific changes in the optic nerve head region and loss of vision.

Since one major risk factor is the occurrence of an elevated intraocular pressure (IOP), and since IOP is substantially regulated by the aqueous outflow pathway tissue, a general classification of different types of glaucoma relates to clinical and morphological findings in the anterior chamber angle region. In human eyes, the most common form is the primary open angle glaucoma (POAG) of the aged adult. Other primary forms include closed angle glaucoma and congenital forms of glaucoma, although this concept is softened with the recognition of a multitude of so called ‘secondary’ glaucomas due to various definable initial events.

## I ANIMAL MODELS USED IN GLAUCOMA RESEARCH

The same classification that is used in human eyes has been introduced for animal models of glaucoma and ocular hypertension. In a review on anterior segment differences by Chew [1], glaucoma was subdivided into inherited, which is rare in all animals except the anterior chamber dysgenic syndromes, congenital, and induced glaucoma, the latter mentioned being the most frequently used group in animal glaucoma research. Induced glaucoma considers the fact that substantial elevated IOP leads to glaucomatous changes in the posterior eye segment. The animals used in these studies are mostly mammals, including mouse, rat, rabbit, pig, dog, cat, and monkey, or birds, including chicken and quail. Although most animals show changes that were considered to be comparable to a glaucomatous situation, it is difficult to correlate these findings with the human situation. One issue in this respect is the different anatomy and morphology not only in the anterior eye segment but also in the lamina cribrosa region and in the inner layer of the retina. To help judging the research findings in these models the present paper reviews the different anatomical situations in the above mentioned animals presently being used in glaucoma research.

## II COMPARATIVE ANATOMY OF THE LAMINA CRIBROSA (LC)

Although numerous species develop a LC (Table **1**), one of the most widely used animal model, the mouse, does not develop connective tissue bundles through the optic nerve head at the level of the sclera [2-5]. This finding is independent of the different mouse strains analyzed. In the rat with an optic nerve head diameter at the level of the sclera only slightly larger than that of the mouse, single collagen bundles are present forming a lamina-cribrosa like structure. The quantity of the LC at the level of the sclera seems dependent on the different strains: a substantial LC was reported in the Brown Norway rat [6] and in the Long Evans rat [7], whereas the PVG Hooded rat [8] and Wistar rat [9] seem to contain only sparse LC bundles.

The lack of the LC in the mouse cannot be explained solely by size-dependent mechanical properties since species with much larger optic nerve head diameters, but myelinated axons reaching into the nerve fiber layer of the retina, also show only a sparse LC. This group of animals includes the rabbit [4,10], quail, and chicken [4]. In these species, the optic nerve head contains neuronal tissue and astrocytes in addition to oligodendroglia cells [11-13].

A multi-layered LC with close three-dimensional similarities to the primate LC is described in the pig [14], cat [4,15,16], and dog eye [17]. The size of the LC diameter (Table **1**) and the variability of the single pores within the LC are comparable in all three species and, again, match the situation in the primate.

## III COMPARATIVE COMPOSITION OF THE LAMINA CRIBROSA

As only qualitative studies are present to date, the comparison of various lamina cribrosa components between species is suggestive.

Immunohistochemical analysis of the extracellular matrix composition of the laminar beams within the LC shows the presence of collagen types I, III, VI, and elastin in the beams, and laminin and collagen type IV (basal membranes) at the border to the astrocytes and around the vessels. This composition is so far only studied in the rat [6,9], monkey [18-22] and human [23-28]. In addition to these electron-microscopically viewable components, chondroitin and dermatan sulfate proteoglycans were localized in the rat [6], monkey [29] and human LC [30,31].

Although the observations warrant further studies utilizing more quantitative techniques, the description in the amount of collagen type VI varies in different species studied: whereas only weak collagen type V and VI is described in the normal human LC [27], intense staining for collagen type VI is documented for the normal rat [6,9] and dog LC [32].

Unfortunately there is a complete lack of data on the composition of the LC in the other animals used for glaucoma research including rabbit, pig, cat, quail, and chicken.

## IV COMPARATIVE ANATOMY OF THE CENTRAL RETINAL VESSELS (TABLE 2)

In rodents (mouse, rat), the central retinal artery (CRA) is derived from a branch of the ophthalmic artery prior to its ramification into the posterior ciliary arteries. A v-shaped intra-arterial cushion is regularly present in the ophthalmic artery just before the branching of the CRA that might influence the vascular flow in this specific region [5,33,34]. The CRA runs towards the sclera and enters the optic nerve obliquely at the level of the sclera and choroid towards the center of the ONH where it branches further forming the retinal arteries. The central retinal vein (CRV) runs closer to the optic nerve than the artery and is connected with the pial venous system [5,35,36].

A special situation is present in the rabbit eye that shows an incompletely vascularized retina restricted to the myelinated portion of the nerve fiber layer [37]. Posterior to the sclera, two to three posterior ciliary arteries form an incomplete arterial circle from which one CRA araises [38]. The venous drainage, in contrast, does not form one main vessel but several branches leaving the retina at the periphery of the ONH. Two prominent veins leave the retina at the nasal and temporal side of the ONH, whereas the superior and inferior branches are much smaller [38].

An almost complementary arrangement of the large retinal vessels described in the rabbit is seen in the pig and dog, where both animals possess a holangiotic retina. In these species, a circulus arteriosus is present around the optic nerve forming several choroidoretinal arteries. From these vessels, up to 6 branches enter the optic nerve head at the level of the sclera and run lateral in the ONH towards the retina [39]. There is no formation of a single CRA. The retinal veins, however, drain the deoxygenated blood towards the center of the ONH forming one CRV that leaves the eye through the LC region [39,40].

In the cat eye, the arterial supply of the retina is similar to that described for the pig and the dog [41-43]: several cilioretinal arteries send branches to the retina in the lateral portion of the ONH. In contrast to the pig and dog, the main retinal veins in the cat eye do not unite in the center of the ONH but leave the eye parallel to the arteries as separate vessels at the lateral portion of the ONH.

Birds (chicken, quail) have an avascular retina which receives its oxygen by a unique vitreal blood vessel aggregation called a pecten. The vessels within the pecten show typical characteristics seen also in retinal and brain vessels by forming a tight-junction barrier [44, 45]. The vessels supplying the pecten run lateral of the ONH and consist of several arterial and venous branches [46, 47].

The central retinal vessels of the primate arise from one CRA and one CRV. The CRA branches from the ophthalmic artery and enters the optic nerve posteriorly to the LC. The CRV runs parallel with the artery through the LC. Both vessels branch in the center of the ONH forming the main retinal vessels.

## V COMPARATIVE ANATOMY OF THE OPTIC NERVE HEAD BLOOD SUPPLY

Due to the difficulties of physiological measurements in this specific region, the data presented is based on corrosion cast preparations and serial sections through the optic nerve head region. Although the number and size of the vessels might indicate the higher or lower importance of the source forming the microvasculature in the optic nerve head region, the precise physiological and patho-physiological role remains hypothetical. The differences between the species are summarized in Table **3**.

In the mouse, both corrosion cast preparations and serial sections revealed that the supply of the optic nerve head is exclusively from recurrent branches of the retina [5]. Neither the choroid nor the pial vessels contribute to this region. In the rat, most of the vessels in the optic nerve head emanate from the retina, too, but some infrequent branches were also observed deriving from the pial vessels [33,34]. There is conflict data regarding vessels in the optic nerve head region deriving from the choroid using corrosion cast preparations: some authors observed branches [35] while others denied their existence [36]. Semithin serial sections through the optic nerve head of Wistar rats did not show vascular branches originating from the choroid but some branches from the posterior ciliary arteries prior to their branching in the choroid (own unpublished data). Further comparative studies are needed to clarify if the different observations are due to different rat strains investigated or due to different methodologies.

A completely different supply of the optic nerve head is observed in the rabbit: most of the smaller vessels show clear arterial and venous connections with the choroid [38]. Next to branches from the arterial circle forming the pial vessels, single branches derive from the central retinal artery within the optic nerve head [38].

Pig, dog and cat show a remarkably similar supply of the optic nerve head region: since they do not possess a single central retinal artery but rather several branches deriving from a plexus of cilioretinal arteries, the main vessels derive from the pial vessels which are in direct contact with the cilioretinal vascular plexus, and from choroidal vessels of the same source [39, 41-43]. The central retinal arteries and the retina are not involved in the supply of the optic nerve head even in the innermost layer towards the retina (in detail only shown for the pig optic nerve head [39]).

In birds (chicken, quail), no data exists about the fine vascular supply of the optic nerve head region.

The vascular supply of the optic nerve head in primates was first introduced by Hayreh [48] using corrosion cast preparations of cynomolgus and rhesus monkeys. In his scheme of arterial blood supply he highlights the influence of all three sources, namely retinal, choroidal, and pial arteries [48]. If the choroidal arteries play the same role in the human eye is not definitely answered: some literature exists on human eyes that questions the involvement of the choroid [49]. The retrolaminar optic nerve in non-human primates and human is mainly supplied by branches deriving from the arterial ‘circle’ of Haller and Zinn formed by anastomoses of the short posterior ciliary arteries [50,51].

## VI COMPARATIVE ANATOMY OF THE GANGLION CELL LAYER (TABLE 4)

Although the gross morphology of the retinal layers is the same in all animals used for glaucoma research, clear differences are present in the inner retina, mainly in the appearance of the ganglion cell layer (GCL). As a general feature in all retinae, ‘displaced’ amacrine cells are present in the GCL with different relative numbers compared to the ganglion cell counts. Their specific role within the GCL has not yet been determined but their inter-species existence might point to a specific role in the GCL and thus upgrade their role as purely ‘displaced’. Since accurate determination of the functional differences between the different amacrine cell classes is difficult, their neurohistochemical profile is required for a more comprehensive explanation of their role.

Most profound variations in the number of ganglion cells are present in different mouse strains: they vary between 32,000 and 87,000 [52]. This strain-variation is less pronounced in the rat, but differences between albino (more than 100,000) and pigmented animals (72,000) were also reported to be significant [53,54]. However, different studies on pigmented animals exist in the literature that might lead to the conclusion that the total number of ganglion cells among different rat strains is more consistence at around 90,000-120,000 [55-58]. Both rodents not only contain ganglion cells in the GCL but numerous displaced amacrine cells, which make up to 59% in the mouse [59] and 50% in the rat [60,61].

In the rabbit retina, one profound finding is the restriction of the vessels to the vascular streak and an otherwise avascular retina. This also leads to a restriction of astrocytes in the retina: they are only present in the myelinated region of the vascular streak [62]. The number of ganglion cells in different albino strains varies between 291,000 [63] and 394,000 [64] showing an accumulation in the area centralis [65] and is comparable to pigmented animals (250,000 – 270,000 [66]). The number of displaced amacrine cells in the GCL of rabbits is lower than in rodents at around 31,7% [67,68].

More pronounced than in the rabbit, the retina of birds (chicken, quail) is completely avascular. A specific glia-cell barrier (peripapillary glia) prevents the vessels from entering into the retina [69]. Similar to the rabbit, partly myelinated axons are localized in the nerve fiber layer in both the quail and the chicken retina [70,71]. Subsequently, oligodendrocytes are located throughout the avian retina with a distinct central-to-peripheral gradient [70,72]. The number of ganglion cells in the avian retina is high and is about twice that of primates (quail: 2,000,000 ganglion cells [73]; chicken: 2,400,000 ganglion cells [74]). In contrast to mammals, there is contrary report about the presence of displaced amacrine cells in the GCL of the bird retina. While almost no amacrines were reported in the quail [73], up to 35% of all cells in the GCL were reported to be amacrine in the chicken [75,76].

The number of retinal ganglion cells in the pig (442,629 cells [77]) and the number of displaced amacrine cells in the GCL (31% [78]) is comparable to that in the rabbit. In contrast to the latter, however, the pig has a holangiotic retina [39] and no intraretinal myelinization of axons. The distribution of astrocytes in the porcine inner retina is similar to that observed in the human eye [79], the cells firmly ensheathing the vessel circumference [80].

In the two carnivores, the number of retinal ganglion cells is 148,303 in the dog [81] and 193,000 in the cat [82]. In comparison to the eye size, the density of ganglion cells in the dog and cat eye is low. However, both species develop an area centralis with accumulation of retinal ganglion cells (dog [83,84], cat [85,86]). In addition, the cat is the most intensively studied animal in regards to retinal ganglion cell differentiation [87-97] and their projections [98-101]. In the dog, astrocyte density varied according to retinal topography with an increased number around retinal blood vessels and in the peripapillary retina [102]. In contrast, astrocyte distribution in the cat retina seemed to be pronounced around the axon bundles of the nerve fiber layer and less intense around blood vessels [103-105]. Displaced amacrine cells in the dog seem to form a homogenous pattern throughout the retina [106]. In the cat, numerous micro-neurons were described in the GCL [107] exceeding the number of ganglion cells fivefold (730,000 - 850,000 displaced amacrine cells [108,109]) and thus representing some 80% of all neurons in the GCL.

In the primate, the number of retinal ganglion cells is comparable to the human eye (cynomolgus monkey: 900,000 – 1,400,000 ganglion cells [110], cercopithecus: 1,228,646 ganglion cells [111], rhesus monkey: 1,500,000 ganglion cells [112], cebus monkey: 1,340,000 – 1,400,000 ganglion cells [113], human: 700,000 – 1,500,000 ganglion cells [114]). Displaced amacrine cells in the GCL show a distinct distribution pattern representing 5-50% of the neurons in the different regions [115]: in the fovea region (-3mm), the number of displaced amacrines is at the lower end (5% of all neurons), whereas more nasally the amount raises up to 30% and temporally up to 50%. The estimations for the human retina are 3% displaced amacrines in the fovea region and up to 80% of displaced amacrines in the peripheral retina [114].

## VII CONCLUSIONS

Several implications can be drawn for the different animal models used in glaucoma research that should be kept in mind when using these species.

The mouse seems to be a good comparative animal model to study the influence of the LC on the process of glaucomatous optic nerve head changes. Since it lacks a LC, the mouse can not be used for LC specific investigations; in addition, the size of the eye leads to different physical and physiological conditions which limit the transfer to the human situation. Strain differences seem to play a crucial role when comparing quantitative data of the optic nerve and retina of different mouse strains. The composition of the optic nerve head is comparable to the human situation (only non-myelinated axons and astrocytes) although the blood supply shows clear differences.

In the rat, strain differences seem to play an important role when investigating the role of the LC. So far, there exist no quantitative studies comparing the LC in different rat strains but the findings in the literature imply differences in susceptibility to elevated pressure between different strains. Such studies could also clarify the role of the LC and its composition on the process of glaucoma. In rat as in mouse, the vascular supply and the localization of the central retinal vessels should be taken into account when comparing findings with the human situation.

The rabbit, chicken and quail seem to be less useful models to study the pathogenic process of glaucoma. The major problem comprises the myelinization of the axons penetrating through the sparsely developed LC into the nerve fiber layer of the retina changing profoundly the situation of cell composition and mechanical reactivity in the optic nerve head region. In addition, the retina is avascular which probably has a major influence on the optic nerve head blood supply, too.

Due to the size and anatomy of the optic nerve head and inner retina, the pig eye has numerous advantages compared to the animals discussed so far. It contains a well-developed lamina cribrosa (as do cat and dog), and the number of retinal ganglion cells is fairly high. The pig has only a poorly developed area centralis, but the possible influence of the fine retinal structure on glaucoma pathology has not yet been evaluated. In contrast to the porcine anatomy, the dog and cat eyes show relatively low values of retinal ganglion cell numbers although the centralization of the retina is more developed showing a clear area centralis. One major advantage to use cat eyes in glaucoma research is the well established classification of retinal ganglion cells. On the other hand, cats (as dogs) have an elaborated tapetum lucidum, which possible might cause trouble when comparing electrophysiological data *in vivo*.

The latter restriction holds also true for the primate monkey eye which shows closest relation to the human anatomy. This might be especially of interest when discussing the role of vascular disturbances and their possible role for the onset and progress of glaucoma.

## VIII SUPPLEMENT: VARIETY OF CURRENT GLAUCOMA MODELS

Rodents. Numerous different glaucoma models exist in the mouse and rat eye comprising almost all aspects of mutations (natural and induced) and manipulations (blood flow changes, intravitreal injections, optic nerve injury). All models were described recently in an excessive review [116].

Rabbit. Corticosteroid-induced ocular hypertension. Intraocular alpha-chymotrypsin injection. Short time intraocular pressure elevation by needle injection.

Pig. Episcleral vein cauterization. *In vitro* models with porcine cadaver eyes (e.g. anterior chamber perfusion).

Dog. POAG in Beagles, American Cocker Spaniels, and other races. Spontaneous secondary glaucoma.

Cat. Primary and secondary narrow angle/ angle closure glaucoma in different cat strains. Short time intraocular pressure elevation by needle injection. Corticosteroid-induced ocular hypertension.

Monkey. A small colony exists with an incidence of natural occurring glaucoma. Manipulations include laser treatment of the trabecular meshwork and damage of the optic nerve.

Chicken. Light-induced glaucoma.

Quail. Natural occurring glaucomatous mutant in the Japanese albino quail.

## Figures and Tables

**Table 1:** Occurrence and Distribution Differences in the Lamina Cribrosa (LC) of Animals Used in Glaucoma Research

	Presence of LC	Diameter of Optic Nerve at the Level of the Sclera/LC	Differences in Composition to the Human [27]
Mouse [2-5]	Ø	193 ±8µm	
Rat [6-9]	(+)		more collagen type VI
Rabbit [4,10]	(+)		
Quail, Chicken [4]	(+)		
Pig [14]	+	1624 ±15µm	
Cat [4,15,16]	+	1187µm	
Dog [17,32]	+	1592µm	more collagen type VI
Monkey [18-22]	+	1717 ±21µm	

Ø = not present; (+) = faintly present; + = well developed.

**Table 2:** Variation of the Central Retinal Vessels – Number and Location at the Optic Disc

	Main Retinal Artery	Main Retinal Vein	
Mouse [5]	1 central	1 central	enters the ON at the level of the sclera
Rat [33-36]	1 central	1 central	enters the ON at the level of the sclera
Rabbit [37,38]	1 central	4 peripheral	
Quail, chicken [44-47]	avascular retina		vessels to the pecten
Pig [39]	6 lateral	1 central	
Cat [41-43]	4-5 lateral	4-5 lateral	
Dog [40]	4-5 lateral	1 central	
Monkey [48]	1 central	1 central	enters the ON retrolaminar
Human [50,51]	1 central	1 central	enters the ON retrolaminar

**Table 3:** Blood Supply of the Optic Nerve Head Region

	Central Retinal Artery	Choroid	Pial Vessels
Mouse [5]	+	Ø	Ø
Rat [33,34]	+	Ø	(+)
Rabbit [38]	(+)	++	+
Quail	n.e.	n.e.	n.e.
Chicken	n.e.	n.e.	n.e.
Pig [39]	Ø	+	++
Cat [41,42]	Ø	+	++
Dog [43]	Ø	+	++
Monkey [48-50]	+	+	+
Human [51-55]	+	(+)	+

n.e. = not examined; Ø = no, (+) = possible, + = clear, ++ = substantial source of optic nerve head capillaries.

**Table 4:** Comparative Data of the Inner Retina

Animal	Ganglion Cell Number (Average)	% of Amacrine Cells of the Ganglion Cell Layer Neurons	Astrocytes in the Inner Retina
Mouse	32,000 – 87,000 [56]	59% [59]	+
Rat	72,371 - 113,000 [52-58]	50% [60,61]	+
Rabbit	250,000 - 394,000 [63,64,66]	31,7% [67,68]	(+), only in vascular streak region [62]
Quail	2,000,000 [73]	? [73]	Ø [69]
Chicken	2,400,000 [74]	- 35% [75,76]	Ø [69]
Pig	442,629 [77]	31% [78]	+, mainly around vessels [79,80]
Cat	193,000 [82]	80% [107-109]	+, mainly around nerve fibers [103-105]
Dog	148,303 [81]		+, mainly around vessels [102]
Monkey	900,000 – 1,500,000 [110-113]	Fovea 5%, nasal 30%, temporal 50% [115]	+, mainly around vessels
Human	700,000 – 1,500,000 [114]	Fovea 3%, peripheral - 80% [114]	+, mainly around vessels

? = No quantified or estimated data available.

Ø = no, (+) = limited, + = ubiquitous presence of astrocytes.
